# High-Dynamic-Range Absorption Spectroscopy of Polymer Sheets and Slabs by Integrating Sphere Illumination and Detection

**DOI:** 10.3390/s26134031

**Published:** 2026-06-25

**Authors:** Juan Carlos Martinez-Anton

**Affiliations:** Facultad de Óptica y Optometría, Universidad Complutense de Madrid, 28037 Madrid, Spain; jcmartin@ucm.es

**Keywords:** absorption spectroscopy, Vis-NIR (400–2500 nm), HILF, polymeric films, integrating sphere, absorption coefficient, PC, PS, PMMA

## Abstract

The optical absorption coefficient *α* of a homogeneous medium is typically determined by measuring the regular transmittance of a plane-parallel optical structure (e.g., a solid sheet or slab). Especially for low absorption, this technique is unsatisfactory as it depends on precise knowledge of the reflectance of the slab/sheet’s faces and/or it needs a very large thickness. Using a specially designed integrating sphere for full-field illumination and detection, we eliminate the dependency on the knowledge of reflectance and increase the sensitivity orders of magnitude at very low absorption. We describe this method analytically and validate it experimentally by comparing the proposed method to the regular ones for different polymeric sheets and slabs. We measure the absorption coefficient of polystyrene (PS) and polycarbonate (PC) sheets and polymethylmethacrylate (PMMA) slabs in the spectral range of 400–2500 nm. With the presented integrating sphere procedure, the absorption coefficient *α* can be reliably obtained, covering four orders of magnitude in a single sample measurement.

## 1. Introduction

The determination of a material’s absorption coefficient *α* or equivalently the extinction coefficient *k* = *αλ*/4π is usually based on measuring the transmittance *T*(λ) of optically homogeneous, plane-parallel optical structures like sheets or slabs [1,2]. Losses in the transmitted flux are not only due to absorption within the material but also to reflection off the sheet faces. For low absorption, these reflection losses need to be corrected with an appropriate model to obtain reliable results. These corrections can be estimated from the Fresnel equations [1,2,3,4,5] if the surfaces are optically polished and the refractive index *n*(*λ*) is known. If we add a reflectance measurement *R*(*λ*) or different sample thicknesses, it is possible to extract the complex refractive index *N* = *n* + i*k* on the assumption of “Fresnel surfaces”, i.e., mathematically flat [5,6,7,8,9,10,11,12,13,14,15]. However, for materials where absorption is extremely small, these methods are unsatisfactory, as the uncertainties are still dominated by the reflection contribution or the assumption of perfect surfaces among other factors. It is known that very slight surface roughness provokes a relevant effect in the estimation of surface reflectance in disagreement with Fresnel equation expectations [16,17,18]. Other alternatives to extract very low absorption coefficients are based on a laser heating a sample and producing a measurable effect like in laser calorimetry, photothermal lens or deflection spectroscopy. These are by nature monochromatic and relatively sophisticated to set up [19].

In this work, we present a spectroscopic measurement method that overcomes dependency on reflection losses knowledge and provides a greater dynamic range for determining the absorption coefficient, especially for very low absorption. Based on a single measurement, the key is to illuminate a plane-parallel sheet or slab from all directions in a uniform way by means of a homogeneous and isotropic light field (HILF). This notoriously simplifies the analytic model, as can be seen in Reference [20]. For example, for low absorption, the flux absorbed by the sample is just proportional to αV where V is the volume of the sample and α its absorption coefficient. The instrumental implementation uses an integrating sphere but adapted to achieve a HILF, as is described in Section 3. Comparatively, a common use of an integrating sphere is to illuminate the sample by impinging it with an external collimated beam and then, the integrating sphere typically collects the reflected and transmitted light (specular or scattered) and finally detects it. Our approach explicitly avoids this type of collimated illumination by spreading the incident light and avoiding direct illumination of the sample [20]. For detection we follow a similar inverted scheme. Here, we have developed a specific analytic model for films and slabs based on the previous contribution [20]. To test the proposed procedure, we measure some polymeric sheets and slabs and compare the results with two regular transmittance techniques. Starting from the same sample, the threshold or the detectable absorption coefficient is at least an order of magnitude lower with the new technique.

## 2. Methods for Extracting the Absorption Coefficient

### 2.1. Classic Transmission Model with Incoherent Illumination

**Figure 1:**
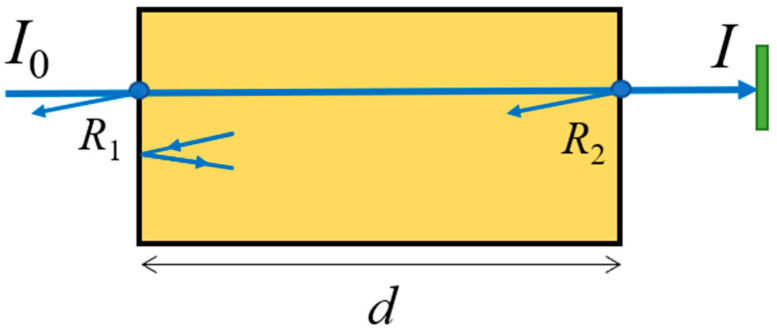
Light interaction scheme in a plane-parallel optical slab of thickness *d*. The two interfaces have a reflectance of *R*_1_ and *R*_2_ respectively. Typically, the reflectance of the faces is assumed to be symmetric and equal, i.e., *R*_1_ = *R*_2_ = *R*_0_. Multiple internal reflections should be considered in total flux transmission.

Absorption spectroscopy is based on the law of attenuation of radiation flux versus the propagation distance *z* of a collimated light beam in a homogeneous medium, typically expressed as

(1) $$I(z)=I_{0}\exp\left[-\alpha \left(\lambda \right)\cdot z\right],$$

where *I* represents the intensity (W/m^2^) versus distance *z*, *I*_0_ is the reference intensity (*z* = 0), and *α*(*λ*) is the absorption coefficient of the medium and depends on the wavelength *λ*. In practice, the medium to be characterized is situated between two optically polished plane-parallel surfaces. A material plate or a polymer film typically meets these conditions. The transmittance *T* of the plate is defined by *T* = *I*/*I*_0_ (Figure 1) and, in a measurement, *I*_0_ may be obtained with no sample and *I* with interposed sample. In this case, we must modify Equation (1) by [5,19,21,22,23]

(2) $$T=\frac{I}{I_{0}}=\frac{\left(1-R_{1}\right)\left(1-R_{2}\right)}{\left[1-R_{1}R_{2}\exp\left(-2\alpha d\right)\right]}\exp\left[-\alpha d\right],$$

where *R*_1_ and *R*_2_ are the reflectance of each face, which depend somewhat on the wavelength *λ*, and *d* is the plate thickness. This is the incoherent model in which we assume that there is no observable interference. In practice, for a freestanding polymer film, the main factor that conditions the observation of interference in the spectra is thickness non-uniformity Δ*d* within the light beam aperture. We will have observable interference approximately for Δ*d* < *λ*/4*n* (~0.4 μm for λ = 2.5 μm and *n* = 1.5). For an average film of *d* = 100 μm, this means that a Δ*d*/*d* > 0.5% will smear out the interference [24]. The denominator term in Equation (2) represents the contribution of multiple reflections to transmission and in practice is very close to 1. As absorption increases, the denominator term tends to 1. At a very low absorption (*αd*→0) and considering *R*_1_ = *R*_2_ = *R*_0_, Equation (2) can be simplified to [21,22,23]

(3) $$T=\frac{I}{I_{0}}=\frac{\left(1-R_{0}\right)}{\left(1+R_{0}\right)}\exp\left[-\alpha d\right]$$

Therefore, we may get the absorption coefficient with

(4) $$\alpha \left(\lambda \right)\cdot d=-\ln\left(T\right)-\ln\left(\frac{1+R_{0}}{1-R_{0}}\right).$$

For increasing absorption, the relative error in determining *αd* using Equation (4) (instead of Equation (2)) remains lower than $R_{0}^{2}$ (<0.45% if *n* < 1.7).

Now, suppose we have two samples of different thicknesses (*d*_1_ and *d*_2_), but with the same or very comparable optical quality on their faces. Comparing its transmittance measurements virtually eliminates the dependence on the reflectance of the faces, *R*_0_. In the specific case of two plates of thicknesses *d* and 2*d* (or double), the analysis simplifies to

(5) $$\alpha \left(\lambda \right)\cdot d=-\ln\left(\frac{T\left(2d\right)}{T\left(d\right)}\right).$$

Now, when absorption increases, the relative error in *αd* using Equation (5) remains lower than *R*_0_^2^/4 (<0.12% if *n* < 1.7). This procedure will be our “gold standard” for comparison in the experimental results.

Typically, we will have an uncertainty in *R*_0_, due to roughness (non-Fresnel surface) or to the unknown chromatic dispersion itself (*n*(λ)) or just a certain dissimilarity among surfaces. Based on the model of Equation (4), the uncertainty of the absorption coefficient at low absorption defined by the minimum resolved *α* (or δ*α*) may be expressed as

(6) $$\delta \alpha ≅\frac{K}{d}.$$

Considering Equation (4), we have $K≃\sqrt{{\left(\delta T\right)}^{2}+4{\left(\delta R_{0}\right)}^{2}}$ and for Equation (5) K≃2δT i.e., the factor *K* depends on the uncertainty of the transmittance δ*T* and the uncertainty of the estimated face reflectance δ*R*_0_. A conservative estimate of uncertainties may be δ*T*~0.002 and δ*R*_0_~0.006. For these estimates and assuming a nominal sheet of *d* = 0.12 mm, the value of *α* below which there is no sensitivity is only δ*α*~0.1 mm^−1^ using Equation (4) or, δ*α*~0.016 mm^−1^ in the double-slab procedure of Equation (5).

### 2.2. Absorption Model Under Homogeneous and Isotropic Illumination (HILF)

**Figure 2:**
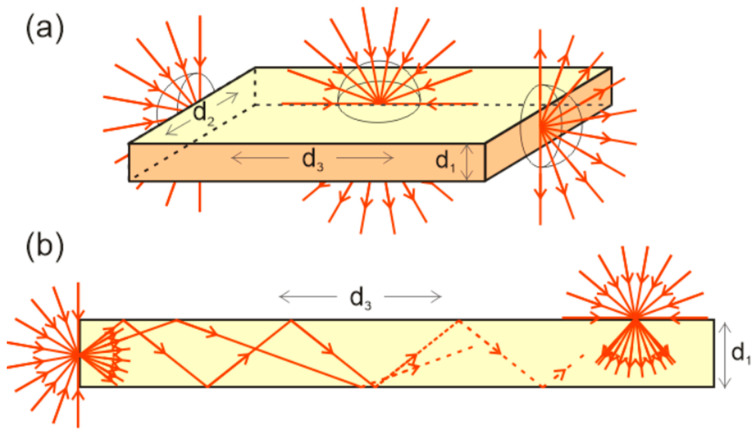
Sample and illumination configuration. (**a**) Slab-like shape (cuboid) makes three independent interactions between opposing faces. (**b**) Light uniformly illuminating a face is internally guided (Total Internal Reflection for *n* > 1.414). Through the thickness *d*_1_, absorption may be very low, while laterally it may be severe (*d*_1_ << *d*_2_, *d*_3_).

The proposed alternative is to measure a sample by illuminating it uniformly from all directions. Instead of illuminating with a collimated beam, we introduce a sample into a homogeneous isotropic light field (or HILF). A special integrating sphere setup will provide such illumination and also permit symmetrical detection [20]. HILF illumination offers significant advantages. Sample location and orientation are not relevant, and, in this situation, we observe that the absorbed light flux is proportional to the object’s volume *V* and to its absorption coefficient *α* when considering low absorption [20]. More specifically, for an infinite sheet of thickness *d*, we may estimate the absorptance *A* of a finite area of one face as the fraction of light absorbed relative to the total incident HILF light on this area. This definition is the same as in the conventional model, and *A* can be estimated as *A* = 1 − *R* − *T*, where *R* represents the reflected light and *T* the transmitted light. Remember that now, the illumination is Lambertian (Figure 2) under HILF. Using an incoherent light model in the reflection and transmission between faces located at a distance *d*, we arrive at the following approximate expression for the absorptance (Equation (29) of [20])

(7) $$A=\frac{1-R_{H}}{1-R_{H}\exp\left(-\alpha \bar{d}\right)}\left[1-\exp\left(-\alpha \bar{d}\right)\right],$$

where *R*_H_ is the hemispherical reflectance of the faces (unpolarized light) and $\bar{d}=2dn^{2}F$ is the mean light path between faces when the illumination is Lambertian. We call the term *n*^2^*F* the optical form factor which, for an infinite sheet, is (Equation (21) of [20])

(8) $$n^{2}F=n^{2}\left(1-\cos\theta_{C}\right)=n^{2}\left[1-{\left(1-\frac{1}{n^{2}}\right)}^{1/2}\right],$$

where *n* is the refractive index of the material and *θ*_C_ is the critical angle (i.e., sin(*θ*_C_) = 1/*n*). Refraction causes the possible paths to approach the normal incidence, and then, the average distance traveled is only slightly greater than the nominal thickness *d*, typically between 1.17*d* for *n* = 1.42 and 1.07*d* for *n* = 2.0. As estimated in [20], the error committed using Equation (7) is less than ~1% over the entire absorption range. Note that in the case of low absorption ($\alpha \bar{d}<∼0.15$), Equation (7) simplifies to $A≅\alpha \bar{d}$ and, therefore, is independent of the hemispherical reflectance *R*_H_.

If the object is a plate or sheet, we may assume the object’s shape as a cuboid, and thus, we can divide the problem into 3 pairs of opposing faces formally equivalent to 3 infinite sheets of different thicknesses *d*_1_, *d*_2_ and *d*_3_ (Figure 2). For *n* > 1.4142, total reflection produces optical guiding of the light, and for practical purposes it behaves as if it were a sheet of infinite extent but limiting its absorption to the area delimited by the faces with areas *S*_1_ = *d*_2_*d*_3_, *S*_2_ = *d*_1_*d*_3_ and *S*_3_ = *d*_1_*d*_2_.

### 2.3. Integrating Sphere’s Measurement Model

In practice, a Lambertian illumination field (isotropic and homogeneous illumination) can be achieved inside an integrating sphere. As described in [20], we get a highly symmetric illumination field, but we also need a symmetrically distributed detection. This ensures that the decentering of a highly absorbing object has a very small impact on the measurement. We know that the irradiance *E*_S_ obtained inside the integrating sphere depends on the introduced flux *ϕ*_0_, the diffuse reflectance of its inner wall *ρ*, the inner area *S*_S_, and the losses through the opening ports at its wall, which are expressed as the ratio between the area of the port *a* (or ports) and the area of the sphere *f* = *a*/*S*_S_, such that

(9) $$E_{S}=\frac{\phi_{0}}{S_{S}}\frac{\rho }{1-\rho \left(1-f\right)}=\frac{\phi_{0}}{S_{S}}M_{0},$$

where *M*_0_ is the so-called amplification factor of the sphere. When we introduce a centered object or body “B”, the absorption in that object can be made almost equivalent to a virtual port whose contribution to the losses we call *f*_B_. Now the average irradiance inside the sphere must be written as

(10) $$E_{B}=\frac{\phi_{0}}{S_{S}}\frac{\rho }{1-\rho \left(1-f\right)\left(1-f_{B}\right)},$$

This is a corrected version of Equation (35) in [20]. The centered body seen from any wall location sometimes may look like an apparent wall-port and sometimes overlaps with a real wall-port. This is the reason why the body loss contribution simply does not add to the other ports as (1 − *f* − *f*_B_) but contributes overall as (1 − *f*)(1 − *f*_B_). If we define the term “spherical transmittance” as *T*_S_ = *E*_B_/*E*_S_, Equations (9) and (10) lead to the following measurement equation

(11) $$\frac{1-T_{S}}{T_{S}}=M_{0}f_{B}.$$

In a measurement, we directly get *T*_S_ and finally obtain the object loss factor *f*_B_. For an object like a sheet or slab, according to Figure 2a, the loss factor *f*_B_ is the sum of the contributions of each pair of facing areas *S*_i_ and can be expressed as

(12) $$f_{B}=\frac{\left(2S_{1}A_{1}+2S_{2}A_{2}+2S_{3}A_{3}\right)}{S_{S}}=\frac{2\left(d_{2}d_{3}A_{1}+d_{1}d_{3}A_{2}+d_{1}d_{2}A_{3}\right)}{S_{S}},$$

and if we apply the known absorptance *A*_i_ from Equation (7), we arrive at

(13) $$f_{B}=\frac{2\left(1-R_{H}\right)}{S_{S}}\left\{\frac{\left[1-\exp\left(-\alpha {\bar{d}}_{1}\right)\right]}{1-R_{H}\exp\left(-\alpha {\bar{d}}_{1}\right)}d_{2}d_{3}+\frac{\left[1-\exp\left(-\alpha {\bar{d}}_{2}\right)\right]}{1-R_{H}\exp\left(-\alpha {\bar{d}}_{2}\right)}d_{1}d_{3}+\frac{\left[1-\exp\left(-\alpha {\bar{d}}_{3}\right)\right]}{1-R_{H}\exp\left(-\alpha {\bar{d}}_{3}\right)}d_{1}d_{2}\right\}$$

After obtaining *f*_B_ in a measurement using an integrating sphere, we find the value of *α* that satisfies Equation (13) for each wavelength. This equation requires knowing the hemispherical reflectance *R*_H_, which we assume to be the same for all faces, and which can be estimated using the Fresnel equations and integrating for all angles in a weighted manner and for unpolarized light. Remember that contrary to the case of direct transmittance (Equation (4)), the knowledge of *R*_H_ is now only relevant in high absorption. For example, at *αd*~3, an uncertainty of *R*_H_ = 0.1 ± 5% leads to a relative error in *α* estimation below ~10%, while at *αd*~0.3 it is below ~0.15%. We have, for *n* = 1.42, *R*_H_ = 0.08, for *n* = 1.5, *R*_H_ = 0.0918, and for *n* = 1.7, *R*_H_ = 0.12.

The detection threshold for the integrating sphere procedure may be derived from Equations ((11)–(13)). For the low absorption limit, we arrive at

(14) $$\delta \alpha \approx \frac{\delta T_{S}}{3.5M_{0}f_{G}d_{1}},$$

where *f*_G_ = *S*_B_/*S*_S_ and *d*_1_ is the minimum thickness. Considering the geometrical ratio *f*_G_ between 0.1 and 0.5, *M*_0_~30, δ*T*_S_~0.002 and *d*_1_ = 0.1 mm, we get a threshold from δ*α*~4·10^−4^ mm^−1^ to 2·10^−3^ mm^−1^, which is one to two orders of magnitude lower than expected in direct transmittance measurements. In Equation (14), we may appreciate that the light multi-pass inside the integrating sphere leads to an effective sample thickness of *M*_0_*d*_1_ and therefore increases the sensitivity.

## 3. Materials and Experimental Results

**Figure 3:**
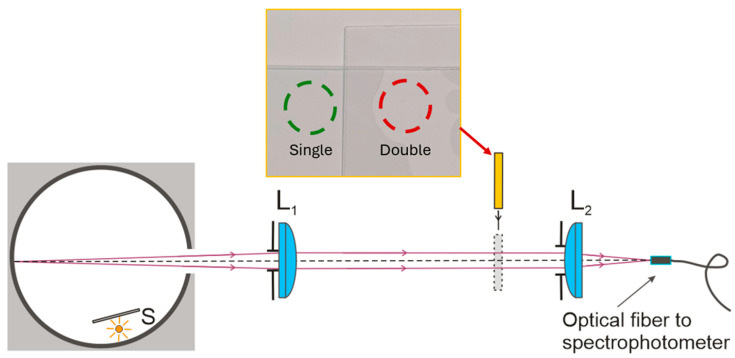
Setup to measure direct transmittance. An integrating sphere serves as a uniform radiance light source that is projected onto a collecting optical fiber by means of two lenses L_1_ and L_2_. The sample is introduced within the light beam. The insert shows a typical sample (PS sheet, 0.51 mm thick) prepared for direct transmission measurement of a single thickness sample (encircled green) and a double thickness sample (encircled red) by means of optical matching of two single sheets.

Figure 3 shows the optical setup to perform direct transmittance measurements. An auxiliar integrating sphere provides a uniform radiance light spot projected with two lenses onto an optical fiber and finally carried to a spectrophotometer. This method has proven highly robust to misalignments of the sample placed within the light path between the lenses (see Figure 3).

**Figure 4:**
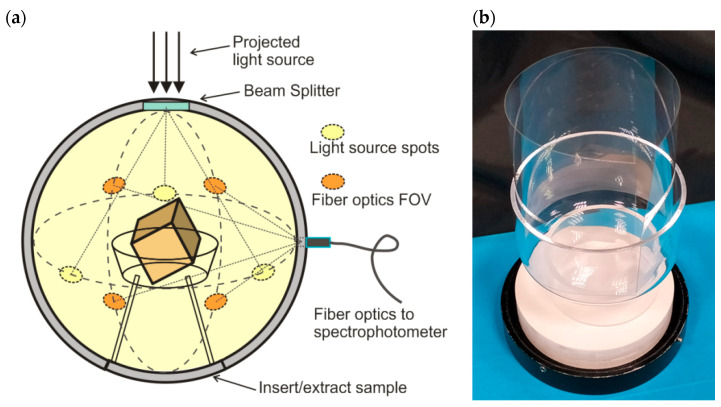
(**a**) Integrating sphere setup. A weakly absorbing quartz holder supports the sample in a centered position. Introduced illumination is projected into three beams spots on the sphere equator. By means of a micro-prismatic beamsplitter an optical fiber collects light by sensing four spots of the integrating sphere wall at a meridian. The illuminating and detection spots are symmetrical around the center to average and reduce the effect of sample decentering and other sample asymmetries. (**b**) Detail of a polymeric sheet sample in its support before it enters the integrating sphere.

Finally, the proposed procedure uses the integrating sphere depicted in Figure 4a and applies Equation (13) to process the data. The integrating sphere provides symmetrically distributed illumination and detection using prisms at the input and output ports (further details may be found in [20]). The sample whose absorption is to be measured is positioned approximately in the center, supported by a basically non-absorbing container [20]. Figure 4b shows a typical sample (PC sheet measuring 170 × 65 × 0.175 mm) before it is introduced inside the integrating sphere.

We measured several polymers in a sheet or plate format to verify the validity of the proposed measurement model. To obtain the absorption coefficient *α*, the measurements were conducted in three ways following the different procedures as explained next. Two methods use direct transmission in normal incidence. The first one applies Equation (4) and the Fresnel equations to calculate *R*_0_ of the faces assuming a constant refractive index (denoted ‘TD.Fresnel’ in the figure legends). The second one uses two different sample thicknesses, a simple sheet or slab and a thicker sample obtained by joining two sheets or slabs just to achieve optical matching. Finally, with these two samples, we may finally apply Equation (5) (denoted ‘DT.DoubleSlab’ in the figure legends).

For both types of measurements (direct transmittance and spherical transmittance), primary light sources are incandescent halogen lamps. A collecting bifurcated optical fiber was connected to a dual-channel spectrophotometer (ARCoptix VIS-NIR-FIB, Arcoptix S.A., Neuchatel, NE, Switzerland ) that covers the spectral range of 350–2500 nm. This instrument integrates two separate modules. A Vis-NIR dispersion grating module covers the range 350–1000 nm (resolution < 1.5 nm). For this module, we have chosen an integration time of 4 ms, a moving average of 2 neighbors and 100 scans. The second module is based on Fourier Transform technology and covers the range 900–2500 nm (resolution of 4 cm^−1^). We take 20 scans at gain set to “high”. Both measurements are sequential and take typically 4 s to complete. At 970 nm the two channel spectra are joined with a proprietary algorithm, and between ~930 and ~1130 nm the signal to noise ratio is low. These instrumental characteristics may explain the higher uncertainty observed in this spectral region for some of the presented results.

Once the apparatus is turned on, we wait about 2 h to limit the temporal shift to below 0.1% within the acquisition time. Overall, the observed uncertainty in the transmittance (regular or “spherical”) is δT~0.2% for the higher signal regions (Vis-NIR).

### 3.1. Sheets of Polycarbonate and Polystyrene

**Figure 5:**
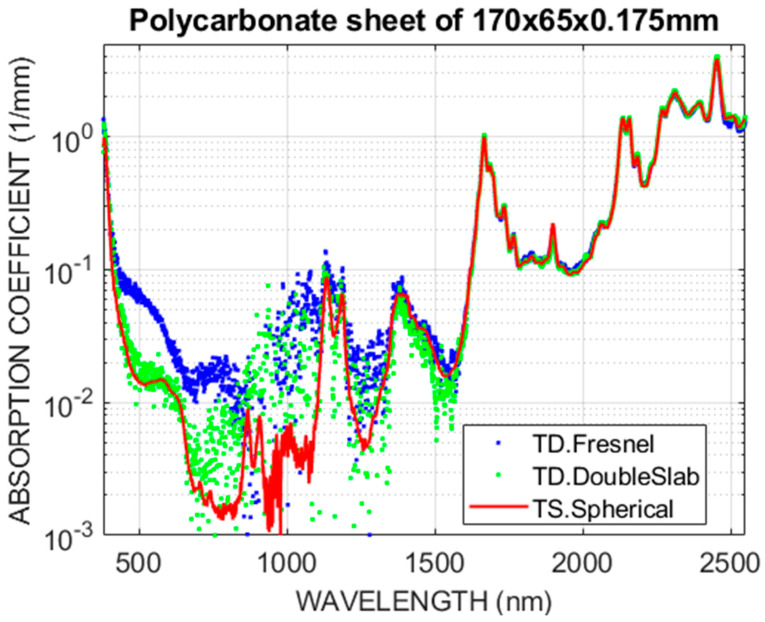
Absorption coefficient for a polycarbonate sheet of 170 × 65 × 0.175 mm estimated with different procedures. Blue dots: direct transmittance corrected of face reflection of *R*_0_ = 0.0485 (Equation (4)). Green dots: direct transmittance of samples of single and double thickness (Equation (5)). Red line: proposed method under “spherical” illumination within an integrating sphere (Equation (13)).

**Figure 6:**
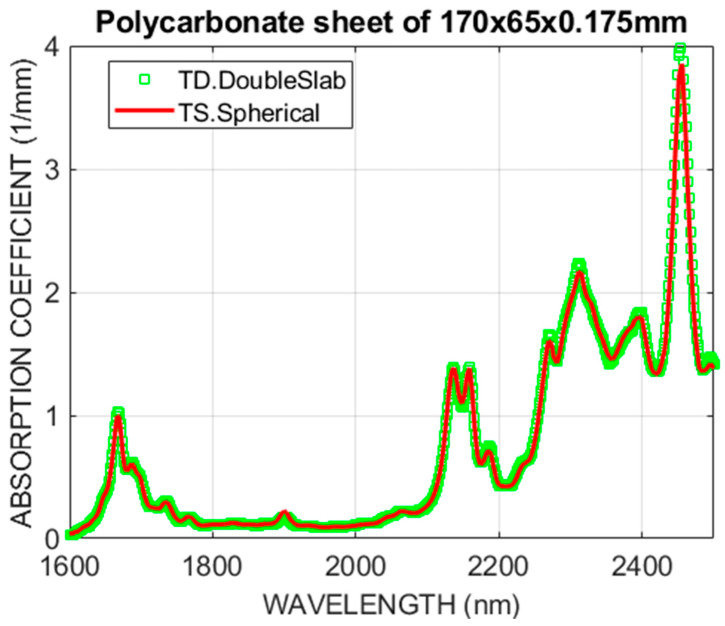
Absorption coefficient for a polycarbonate sheet of 170 × 65 × 0.175 mm. Detail of Figure 5 (abscissas in linear scale) shown for λ > 1600 nm. Comparison of double slab method (green squares) to the proposed method (red line).

**Figure 7:**
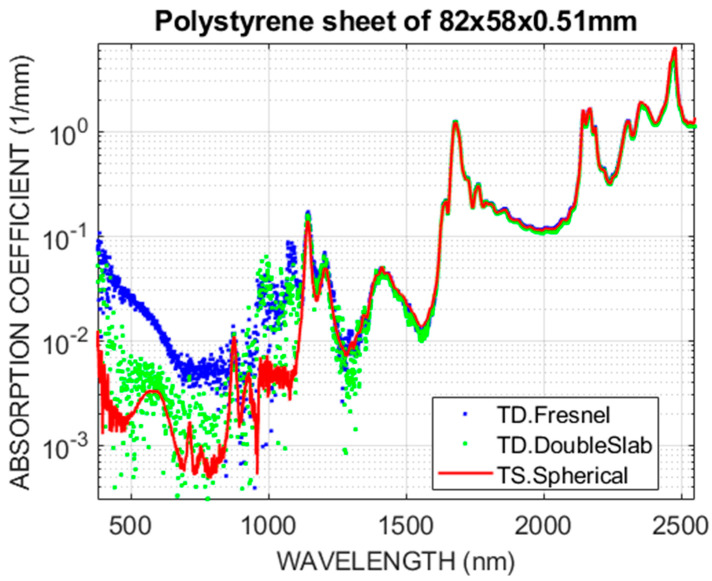
Absorption coefficient for a polystyrene sheet of 82 × 58 × 0.51 mm estimated with different procedures. Blue dots: direct transmittance corrected of face reflection of *R*_0_ = 0.0485 (Equation (4). Green dots: direct transmittance of samples of single and double thickness (Equation (5)). Red line: proposed method under “spherical” illumination within an integrating sphere (Equation (13)).

We selected high-quality commercial polymer films, meaning an excellent surface finish, homogeneity, and transparency. Specifically, we used a 0.175 mm thick polycarbonate sheet (ref. Lexan^TM^ 8010 film 7mil, TEKRA, LLC, New Berlin, WI, USA) and a 0.51 mm thick polystyrene sheet, typically used as framed photo protectors. Figure 5 and Figure 6 show the results of the absorption coefficient estimation for the polycarbonate film, and Figure 7 shows the results for the polystyrene film. The direct transmission measurement results are denoted as ‘TD.Fresnel’ when reflectance estimation *R*_0_ uses Fresnel equations and a constant refractive index (Equation (4)), and denoted as ‘TD.DoubleSlab’ when using the improved double-slab procedure (Equation (5)). This last procedure obtains greater reliability across the entire range. For the first case, the reflection correction applied to both the PC and PS sheets is *R*_0_ = 0.0485, which corresponds to a refractive index of *n* = 1.565 at *λ* = 1.5 μm after [12]. As expected, the results in the visible part deviate from the better procedure of using two thicknesses. In this last procedure, the expected resolution or threshold of *αd*_1_ is δ(*αd*_1_) » δ*T* and for a δ*T*~0.002, we get δ*α*~0.01 mm^−1^ for the PC sheet and δ*α*~0.004 mm^−1^ for the PS sheet, congruent with the figures.

The proposed method with integrating sphere (denoted as ‘TS.Spherical’) performs well across the entire range, with noise well below that of direct transmittance best estimations. This is particularly noticeable in the observed spectral details or resolution achieved in the Vis-NIR region (400–900 nm) where absorption is very low. Figure 6 shows the spectral region of highest absorption (above *λ* > 1600 nm) in linear scale to appreciate how the methods compare in higher absorption, validating the proposed method for that absorption range and by extension also for very low absorption, where direct transmittance methods cease to be adequate.

**Figure 8:**
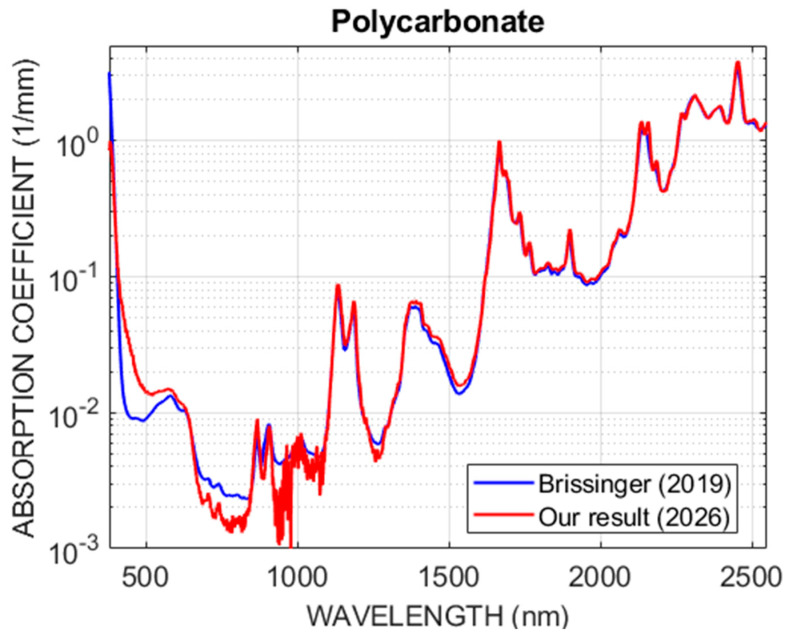
Absorption coefficient for polycarbonate. Comparison between our results (red) and the data of Reference [7] (blue).

Finally, in Figure 8, we compare our results for PC with the data from Reference [7]. In the comparison, we may appreciate discrepancies that may be attributed to instrumental differences and/or different material properties coming from different manufacturing processes (additives, molecular weight, optical homogeneity, etc.).

### 3.2. Slabs of PMMA

**Figure 9:**
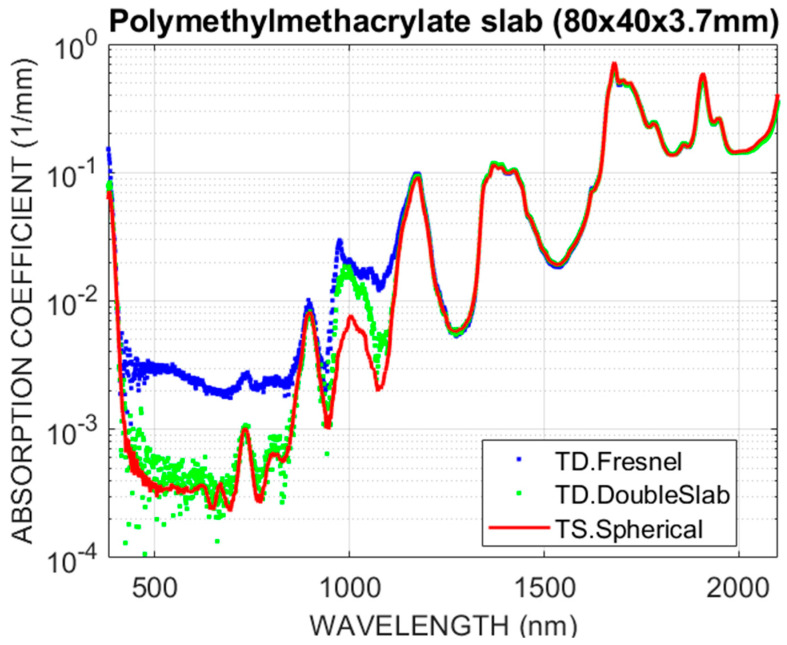
Absorption coefficient of a PMMA slab (80 × 40 × 3.65 mm) estimated with different procedures. Blue dots: direct transmittance corrected of face reflectance (Equation (4)) with Fresnel estimation of *R*_0_ = 0.0365. Green dots: direct transmittance of samples of single and double thickness (Equation (5)). Red line: proposed method under “spherical” illumination within an integrating sphere (Equation (13)).

In this case, we worked with commercial slabs of polymethyl methacrylate (PMMA) measuring 40 × 80 × 3.65 mm and thermally polished at the edges. For a double-thickness slab, we use two single slabs optically coupled by bonding them with a few drops of chloroform, ensuring that the solvent thickness is less than 50 µm so that its impact on the absorption spectrum is negligible. Figure 9 shows the absorption coefficient estimated using the different methods. The results point out similar conclusions to those obtained with the PC and PS sheets, although the direct measurements now have a better resolution for *α* since the thickness *d*_1_ = 3.65 mm is much greater. Now we apply a Fresnel face reflectance correction of *R*_0_ = 0.0365, which corresponds to *n* = 1.472 at *λ* = 1.5 μm, after [12]. The best threshold value estimate in direct transmittance now yields δ*α*~5 × 10^−4^ mm^−1^, in agreement with the observed results in Figure 9, while for the integrating sphere procedure, it achieves δ*α*~4·10^−5^ mm^−1^, as expected from Equation (14). The absorption of the PMMA in the visible spectrum is very low, but the integrating sphere method clearly resolves absorption details with a resolution an order of magnitude better than the double-thickness “gold standard”. Figure 9 also shows that within the spectral range of 930 nm to 1130 nm, the direct transmittance results appear to yield highly distorted values. This is probably due to the low signal-to-noise ratio (SNR) of the spectrophotometer in this spectral range in combination with other instrumental effects that contribute to a distorted evaluation of the absorption coefficient. However, the integrating sphere results do not show this distortion. Higher sensitivity provides a better SNR, and the same instrumental effects do not seem to affect the results as much.

**Figure 10:**
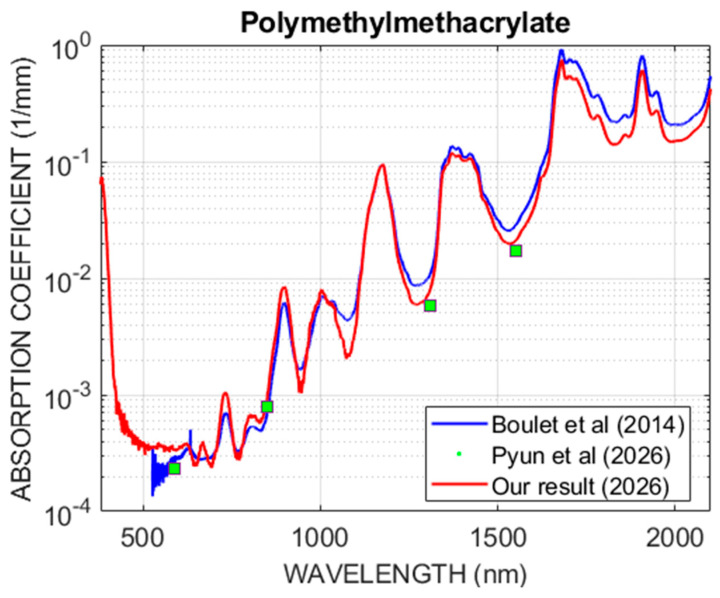
Polymethylmethacrylate absorption coefficient comparison. Blue curve: data from Boulet et al. [25]. Green dots from Pyun et al. [26]. Red curve: our results.

As an alternative comparison, we show in Figure 10 our results for the PMMA and some data from the literature [25,26]. We may appreciate discrepancies that may be attributed to similar reasons as observed for Figure 8. Nevertheless, the overall spectrum does not distort severely over the four-order range.

## 4. Conclusions

Using a special integrating sphere setup to uniformly illuminate a sample, we may obtain the absorption coefficient *α*(*λ*) of a material in a sheet or slab format with significant advantages. We reduce the threshold or the minimum *α* detectable by at least one order of magnitude. This is partially due to the multi-pass effect inside the integrating sphere. In the lower limit of absorption, there is no need of a reflection loss correction as is necessary in some conventional transmittance measurements. We have shown that with a specific integrating design and measurement model we achieve a reliable coefficient of absorption over a high dynamic range with a single measurement and sample. Care in sample alignment is also unnecessary; it can be placed loosely centered within the integrating sphere. These three factors make the proposed technique a good choice compared to conventional techniques based on direct transmittance measurements, especially for materials and thicknesses that contribute little to absorption, as has been shown for polymeric transparent materials in the Vis-NIR. The proposed technique provides a greater resolution at low absorption while maintaining reliability and easiness in a higher absorption range.

## Data Availability

Available upon a reasonable request.
